# Risk of solid cancer in patients exposed to anti-tumour necrosis factor therapy: results from the British Society for Rheumatology Biologics Register for Rheumatoid Arthritis

**DOI:** 10.1136/annrheumdis-2013-204851

**Published:** 2014-03-31

**Authors:** Louise K Mercer, Mark Lunt, Audrey L S Low, William G Dixon, Kath D Watson, Deborah P M Symmons, Kimme L Hyrich, BSRBR Control Centre Consortium

**Affiliations:** Arthritis Research UK Centre for Epidemiology, Centre for Musculoskeletal Research, Manchester Academic Health Science Centre, The University of Manchester, Manchester, UK

**Keywords:** Rheumatoid Arthritis, Anti-TNF, Epidemiology

## Abstract

**Background:**

Patients with rheumatoid arthritis (RA) have an increased risk of certain solid cancers, in particular lung cancer, compared to the general population. Treatment with tumour necrosis factor (TNF) inhibitors (TNFi) may further enhance this risk.

**Objectives:**

To compare the risk of solid cancer in patients with RA treated with TNFi to that in patients treated with non-biologic (synthetic) disease modifying antirheumatic drugs (sDMARDs).

**Methods:**

Patients with a physician diagnosis of RA enrolled in the British Society for Rheumatology Biologics Register, a national prospective cohort study established in 2001 to monitor the long-term safety of TNFi, were followed via record linkage with the national cancer registries until first solid cancer, death, for 5 years, or until 2011. Rates of solid cancers in 11 767 patients without prior cancer who received TNFi were compared to those in 3249 patients without prior cancer treated with sDMARDs.

**Results:**

427 solid cancers were reported in 52 549 patient-years follow-up for the TNFi group (81 (95% CI 74 to 89) per 10 000 patient-years) and 136 cancers were reported in 11 672 patient-years in the sDMARD cohort (117 (95% CI 98 to 138) per 10 000 patient-years). After adjusting for differences in baseline characteristics there was no difference in risk of solid cancer for TNFi compared to sDMARD treated patients: HR 0.83 (95% CI 0.64 to 1.07). There was no difference in the relative risk of cancer for any of the individual TNFi drugs.

**Conclusions:**

The addition of TNFi to sDMARD does not alter the risk of cancer in RA patients selected for TNFi in the UK.

## Introduction

Tumour necrosis factor α (TNF) plays a complex role in the development and progression of tumours.^1–4^ From early in the development of TNF inhibitors (TNFi), there was concern that their use might lead to an increased risk of malignancy in patients with rheumatoid arthritis (RA). Patients with prior malignancy were therefore excluded from the majority of TNFi randomised controlled trials (RCTs). An early meta-analysis of RCTs fuelled concerns that TNFi may increase the risk of cancer, when it reported an almost fourfold increase in solid cancers in patients treated with infliximab (INF) or adalimumab (ADA) versus placebo.^5^ Although a number of subsequent meta-analyses have not replicated the finding,^6^ ^7^ concerns have persisted. Few long-term observational studies have reported on the risk of solid cancer following TNFi use, and no association with an overall increased risk of cancer has been found.^8–12^

The primary aim of this study was to determine the incidence of solid cancer in people with RA treated with TNFi, and to compare this to the incidence in biologic-naïve patients treated with non-biologic (synthetic) disease modifying antirheumatic drugs (sDMARDs). Additional aims were: (i) to examine and compare, where possible, the site-specific risk of solid cancer; and (ii) to compare the survival following diagnosis of solid cancer in patients treated with TNFi versus sDMARDs.

## Methods

### Patients

Patients were participants in the BSRBR-RA, a national prospective cohort study established in 2001 to examine the long-term safety of biologic therapy in RA. Patients starting treatment with one of the first three available TNFi (etanercept (ETA), INF and ADA) were recruited from across the UK. UK guidelines recommend that TNFi use is restricted to patients with active disease (28 joint disease activity score (DAS28)^13^ >5.1) despite treatment with at least two sDMARDs, one of which should be methotrexate.^14^ A comparison cohort of biologic-naïve RA patients, with active disease despite current treatment with sDMARDs (guideline DAS28 ≥4.2), was recruited from 28 sites.^15^ The subjects’ written consent was obtained.

#### Baseline

Baseline data collected via nurse-completed questionnaire included age, sex, RA disease duration, DAS28, current and past sDMARDs, baseline glucocorticoid use, co-morbidities and smoking history. Patients completed a Stanford Health Assessment Questionnaire (HAQ)^16^ to indicate level of physical disability and were asked to select their ethnic group from a list. Previous malignancies, including date and site, were identified via record linkage with the National Health Service Information Centre (NHS IC) and the Northern Ireland Cancer Registry. Capture of cancer cases is very high using these sources, for example 97% for cancers occurring in England in 2009.^17^

#### Follow-up and outcome

All patients were followed in identical manner. Changes to RA therapy were reported on nurse-completed questionnaires 6-monthly for 3 years then annually thereafter. Data on adverse events (including cancers) were captured in three ways: nurse-completed questionnaires; 6-monthly patient health diaries (first 3 years only); and by flagging with the national cancer agencies which reported malignancies using the 10th edition of the International Classification of Diseases (ICD-10). The primary outcome measure was the first verified solid cancer per subject. Solid cancers comprised all cancers except lymphoproliferative or myeloproliferative malignancies and keratinocyte skin cancers. Additional information (including histology) was sought from physicians for all reported cancers, using a standardised proforma. Cancers were verified if they were either confirmed on a histology report or reported by a national cancer agency.

### Statistical analysis

**Figure 1:**
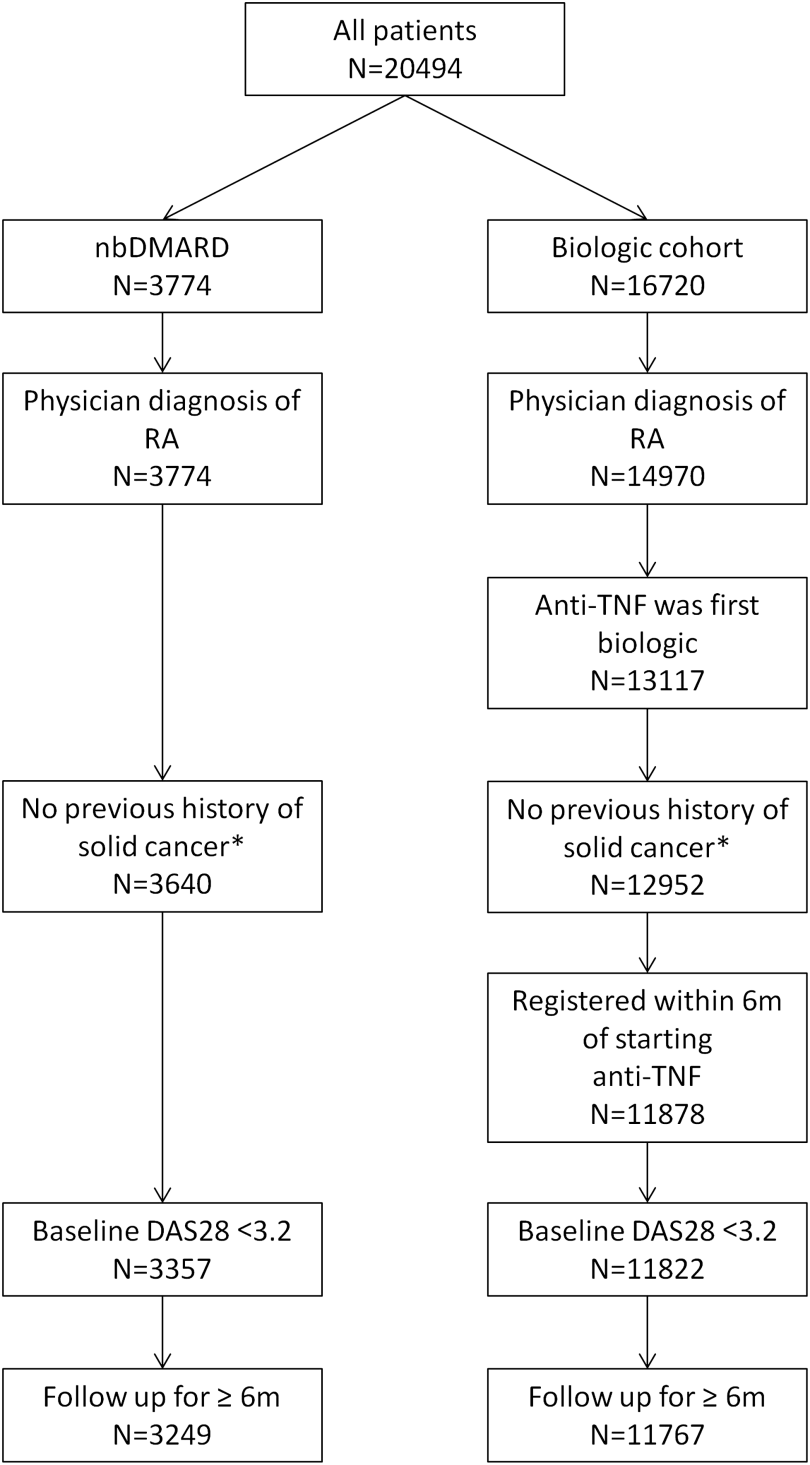
Selection of participants for the analysis. *As reported to the BSRBR-RA by the national cancer registries. DAS28, disease activity score; nbDMARD, non-biologic disease modifying antirheumatic drug; RA, rheumatoid arthritis; TNF, tumour necrosis factor.

The analysis included patients with a physician diagnosis of RA who had at least one returned nurse-completed follow-up questionnaire by 31 January 2011 (figure 1). The TNFi cohort comprised patients who received ETA, INF or ADA as their first biologic therapy with or without concomitant sDMARDs and who had been registered within 6 months of starting treatment. Patients with a diagnosis of solid cancer reported by a national cancer registry prior to TNFi initiation (or study registration in the sDMARD cohort) were excluded. The first 6 months of follow-up time was excluded from both cohorts, to minimise selection bias. Patient-years of follow-up time were calculated from 6 months after the date of starting a TNFi, or 6 months after the date of registration for the sDMARD cohort. Follow-up was censored at the date of diagnosis of the first solid cancer, death, after contributing 5 years of follow-up to the analysis (excluding the first 6 months), or on 31 January 2011, whichever came first. Patients in the sDMARD cohort who subsequently started a biologic drug contributed follow-up time until the first dose of biologic therapy, and subsequent follow-up time to the TNFi cohort if they consented to be re-recruited and the cohort was still recruiting.

Rates of cancer are presented as total events per 10 000 patient-years with 95% CIs. Risk comparisons were made between the TNFi and sDMARD cohorts using Cox regression. Potential confounders were identified a priori and comprised: age; sex; smoking history (current/former/never); ethnicity (dichotomised as white or non-white due to the high proportion of white participants); co-morbidity; DAS28 score; HAQ score; RA duration; number of previous sDMARDs (categorised as ≤3 or ≥4); exposure to glucocorticoids at baseline; prior exposure to azathioprine (AZA); and prior exposure to cyclophosphamide (CYC). Co-morbidity was included as a composite variable constructed from presence of: hypertension; ischaemic heart disease (myocardial infarction and/or angina); stroke; lung disease (asthma, bronchitis or emphysema); renal disease; diabetes mellitus; liver disease; or depression. Registration date with the BSRBR-RA (before/after June 2004) was also included to account for other unmeasured confounding relating to temporal changes in the way that rheumatologists managed patients with RA.

Adjustment for these covariates was performed by calculating a propensity score (PS) which reflected the probability that each patient received TNFi given their baseline characteristics. The PS has a single value for each subject that can be used in the regression model in place of all of the potentially confounding covariates. The balance of the model was tested by examining the likely bias in the treatment estimate due to each confounder. Subjects with low disease activity at baseline (DAS28 ≤3.2) (sDMARD: 283; TNFi: 56) were excluded from the analysis in order to ensure positivity in the PS model. The PS was stratified into deciles (PD). Missing baseline data were replaced using multiple imputation (see online only supplementary methods). The assumption of proportionality was tested using Schoenfeld residuals. Stata V.12.1 was used.

#### Drug exposure models

The primary analysis compared patients ever treated with TNFi to those in the sDMARD cohort—that is, subjects in the TNFi cohort were considered exposed from first dose until the end of follow-up. Four sensitivity analyses were performed. First, time at risk in the TNFi cohort was restricted to time on drug or within 90 days of the first missed dose. Second, cumulative time on TNFi (plus a 90-day lag window) was calculated for every participant in the TNFi cohort. This cumulative exposure time was then categorised into <1.5 years, 1.5–3 years and ≥3 years and compared to sDMARD using Cox regression. For these sensitivity analyses, patients were censored at the date of their last received nurse-completed questionnaire since information about current drug exposure came from these questionnaires. Third, all follow-up time was included, rather than omitting the first 6 months. Finally, an analysis restricted to cancers reported by the cancer registries was performed.

The risk of first solid cancer for each TNFi agent was compared separately to sDMARDs. In these analyses, cancers were attributed to the most recently received TNFi—that is, patients contributed follow-up time to their first TNFi to the point of switching, after which follow-up and cancers were attributed to the most recent drug only. Histology reports and ICD-10 codes reported by the cancer registries were used to determine cancer sites. Site-specific analyses were performed for sites with at least 10 cancers in each cohort.

#### Outcome following cancer diagnosis

Mortality following cancer diagnosis was compared between patients ever exposed to TNFi and the sDMARD cohort by using Cox regression, adjusted for age as a time varying covariate and sex. Deaths were identified by record linkage with the national death registry. Deaths occurring up to 31 January 2012 were included—that is, one year after the last day of follow-up for cancer diagnosis.

## Results

A total of 3249 sDMARD and 11 767 TNFi patients were included (figure 1). The sDMARD cohort was older and comprised more men (table 1). The TNFi cohort had more severe disease of longer duration and greater exposure to glucocorticoids and prior sDMARDs, including AZA and CYC.

**Table 1:** Baseline characteristics of the cohorts

	sDMARD	All TNFi	First TNFi drug		
	N=3249	N=11 767	ETA	INF	ADA
			N=4073	N=3457	N=4237
Mean age: years (SD)	60 (12)	56 (12)	56 (12)	56 (12)	56 (12)
Female: %	2381 (73)	8977 (76)	3150 (77)	2614 (76)	3213 (76)
Smoking history (%)					
Current smoker	770 (24)	2569 (22)	834 (22)	756 (22)	979 (23)
Former smoker	1276 (39)	4466 (38)	1551 (38)	1309 (38)	1606 (38)
Never smoked	1188 (37)	4656 (40)	1661 (41)	1374 (40)	1621 (38)
Not recorded	14 (0)	76 (1)	27 (1)	18 (1)	31 (1)
Ethnicity (%)					
White	2459 (76)	9725 (83)	3380 (83)	2798 (81)	3547 (84)
Other	62 (2)	407 (3)	141 (3)	126 (4)	140 (3)
Not recorded	728 (22)	1635 (14)	552 (14)	533 (15)	550 (13)
Mean DAS28 (SD)	5.3 (1.1)	6.6 (1.0)	6.6 (0.9)	6.6 (1.0)	6.5 (1.0)
Mean HAQ (SD)	1.5 (0.7)	2.0 (0.6)	2.1 (0.6)	2.1 (0.5)	1.9 (0.6)
Median disease duration: years (IQR)	6 (1, 15)	11 (6, 19)	12 (6, 19)	12 (6, 19)	10 (5, 18)
Baseline steroid use: (%)	726 (22)	5190 (44)	1949 (48)	1596 (46)	1645 (39)
Number of prior sDMARDs: median (IQR)	2 (1, 3)	4 (3, 5)	4 (3, 5)	4 (3, 5)	3 (3, 4)
Co-morbidity* (%)					
None	1358 (42)	5466 (46)	1840 (45)	1626 (47)	2000 (47)
1 co-morbidity	1123 (35)	4043 (34)	1388 (34)	1212 (35)	1443 (34)
2 co-morbidities	535 (16)	1662 (14)	616 (15)	470 (14)	576 (14)
≥3 co-morbidities	233 (7)	596 (5)	229 (6)	149 (4)	218 (5)
Year of registration (%)					
Pre-2003	7 (0)	1410 (12)	203 (5)	1177 (34)	30 (1)
2003	279 (9)	2070 (26)	1498 (37)	1102 (32)	470 (11)
2004	752 (23)	3227 (27)	1951 (48)	495 (14)	781 (18)
2005	797 (25)	1613 (14)	418 (10)	346 (10)	849 (20)
2006	668 (21)	1125 (10)	2 (0)	272 (8)	851 (20)
2007	331 (10)	843 (7)	1 (0)	65 (2)	777 (18)
2008–2009	415 (13)	479 (4)	0 (0)	0 (0)	479 (11)

*Hypertension, ischaemic heart disease (myocardial infarction or angina), stroke, asthma, bronchitis or emphysema, diabetes mellitus, depression, renal disease and liver disease.

ADA, adalimumab; DAS28, disease activity score; ETA, etanercept; HAQ, Health Assessment Questionnaire; INF, infliximab; sDMARD, synthetic disease modifying antirheumatic drug; TNFi, tumour necrosis factor inhibitor.

A total of 563 cancers were diagnosed during 64 221 patient-years of follow-up (136 in 11 672 patient-years in the sDMARD and 427 in 52 549 patient-years in the TNFi cohorts) (table 2). More than 90% of cancers in both cohorts were reported by the national cancer agencies. The proportion of cancers reported on the nurse questionnaire was higher in the TNFi cohort (table 2). A further 89 cancers were reported to the BSRBR (sDMARD 22; TNFi 67) but were not verified, and so were excluded from the analysis.

**Table 2:** Association between exposure to TNFi and development of new solid cancer

	sDMARD	TNFi
	N=3249	N=11 767
*Ever-exposed to TNFi model*		
Total follow-up time (patient-years)	11 672	52 549
Follow-up per subject; median (IQR)	4.1 (2.3, 5.0)	5.0 (4.4, 5.0)
Cancers	136	427
Sources of reporting of solid cancers		
Cancer registry (%)	126 (93)	399 (93)
Consultant/nurse (%)	83 (61)	322 (75)
Patient (%)	23 (17)	79 (19)
Incidence rate per 10 000 patient-years (95% CI)	117 (98 to 138)	81 (74 to 89)
Unadjusted HR (95% CI)	Referent	0.70 (0.58 to 0.85)
Age and sex adjusted HR (95% CI)	Referent	0.91 (0.75 to 1.11)
PD adjusted HR (95% CI)	Referent	0.83 (0.64 to 1.07)
On TNFi (plus 90 days)*		
Follow-up time (patient-years)	10 275	39 173
Cancers	106	285
PD adjusted HR (95% CI)	Referent	0.81 (0.60 to 1.10)
*Cumulative exposure to TNFi*		
<1.5 years		
Follow-up time (patient-years)	10 275	20 264
No. solid cancers	106	166
PD adjusted HR (95% CI)	Referent	0.87 (0.66 to 1.15)
1.5 to <3 years		
Follow-up time (patient-years)	10 275	14 729
No. solid cancers	106	99
PD adjusted HR (95% CI)	Referent	0.85 (0.63 to 1.17)
PD adjusted HR (95% CI); <1.5 years referent		0.91 (0.67 to 1.24)
≥3 years		
Follow-up time (patient-years)	10 275	13 969
No. solid cancers	106	100
PD adjusted HR (95% CI)	Referent	0.77 (0.58 to 1.03)
PD adjusted HR (95% CI); <1.5 years referent		0.77 (0.58 to 1.02)
*All follow-up time (including first 6 months)*		
Follow-up time (patient-years)	13 425	58 437
Cancers	166	449
Incidence rate per 10 000 patient-years (95% CI)	124 (106 to 144)	77 (70 to 84)
PD adjusted HR (95% CI)	Referent	0.77 (0.60 to 0.98)
*Cancer registry reported cancers only*		
Follow-up time (patient-years)	11 758	52 549
Cancers	126	399
Incidence rate per 10 000 patient-years (95% CI)	107 (89 to 128)	76 (69 to 84)
PD adjusted HR (95% CI)	Referent	0.86 (0.66 to 1.13)

*Time after last received consultant follow-up form excluded from these analyses.

PD, propensity score stratified into deciles; sDMARD, synthetic disease modifying antirheumatic drug; TNFi, tumour necrosis factor inhibitor.

The unadjusted HR for TNFi compared to sDMARDs was 0.70 (95% CI 0.58 to 0.85) (table 2). Age, male gender, white ethnicity, smoking, co-morbidity, RA severity, prior exposure to ≥4 sDMARDs, and CYC were associated with risk of cancer in univariate analyses (see online supplementary table S2). After fully adjusting, using PD, there was no difference in the risk of cancer for TNFi compared to sDMARD exposed patients (_adj_HR 0.83, 95% CI 0.64 to 1.07; table 2). A total of 285 solid cancers occurred while the patient was actively receiving a TNFi (or within 90 days of the first missed dose). The PD adjusted HR for TNFi was 0.81 (95% CI 0.60 to 1.10). There was no change in the PD adjusted HR with alternative exposure models or when the outcome was restricted to cancers reported by the cancer agencies (table 2). There was also no observed difference in adjusted rates when looking at each TNFi separately (table 3).

**Table 3:** Comparison of risk of individual TNFi therapies with sDMARD therapy

	ETA	INF	ADA
	N=4073	N=3457	N=4327
Follow-up time (patient-years)	22 146	12 379	18 027
Follow-up per subject in years: median (IQR)	4.8 (2.5, 5.0)	3.9 (1.3, 5.0)	3.5 (2.0, 4.8)
Solid cancers	190	98	139
Incidence rate per 10 000 patient-years (95% CI)	86 (74 to 99)	79 (64 to 96)	77 (65 to 91)
Unadjusted HR (95% CI)*	0.74 (0.59 to 0.92)	0.68 (0.53 to 0.88)	0.67 (0.53 to 0.84)
Age and sex adjusted HR (95% CI)	1.00 (0.80 to 1.25)	0.87 (0.67 to 1.12)	0.84 (0.66 to 1.07)
PD-adjusted HR (95% CI)	0.89 (0.67 to 1.19)	0.81 (0.59 to 1.11)	0.79 (0.59 to 1.05)

*sDMARD was referent for regression analyses.

ADA, adalimumab; ETA, etanercept; INF, infliximab; PD, propensity score stratified into deciles; sDMARD, synthetic disease modifying antirheumatic drug; TNFi, tumour necrosis factor inhibitors.

There was no significant difference in proportion or relative risk (RR) for any of the most common site specific cancers (table 4 and see online supplementary table S3), although there was a suggestion of a possible reduction in the risk of breast and colorectal cancers in the TNFi cohort.

**Table 4:** Incidence and risk of individual solid cancer subtypes

	sDMARD	TNFi	ETA	INF	ADA
	N=3249	N=11 767	N=4073	N=3457	N=4327
Lung cancer					
Number	40	103	49	25	29
Incidence rate per 10 000 patient-years (95% CI)	34 (24 to 47)	20 (16 to 24)	22 (16 to 29)	20 (13 to 30)	16 (11 to 23)
Unadjusted HR (95% CI)	Referent	0.57 (0.40 to 0.82)	0.64 (0.42 to 0.98)	0.59 (0.36 to 0.97)	0.49 (0.29 to 0.76)
Age and sex adjusted HR (95% CI)	Referent	0.81 (0.56 to 1.17)	0.95 (0.62 to 1.46)	0.81 (0.49 to 1.35)	0.64 (0.40 to 1.04)
PD-adjusted HR (95% CI)	Referent	0.85 (0.52 to 1.39)	1.02 (0.58 to 1.76)	0.92 (0.50 to 1.71)	0.69 (0.39 to 1.23)
Female breast cancer					
Number	22	73	30	18	25
Incidence rate per 10 000 patient-years (95% CI)	34 (20 to 48)	18 (14 to 22)	17 (11 to 23)	19 (10 to 28)	17 (10 to 23)
Unadjusted HR (95% CI)	Referent	0.72 (0.45 to 1.17)	0.70 (0.40 to 1.22)	0.76 (0.41 to 1.42)	0.74 (0.42 to 1.31)
Age adjusted HR (95% CI)	Referent	0.83 (0.51 to 1.35)	0.83 (0.47 to 1.45)	0.86 (0.46 to 1.61)	0.83 (0.47 to 1.48)
PD-adjusted HR (95% CI)	Referent	0.58 (0.32 to 1.06)	0.56 (0.28 to 1.10)	0.59 (0.28 to 1.24)	0.59 (0.31 to 1.15)
Colorectal cancer					
Number	19	43	16	10	17
Incidence rate per 10 000 patient-years (95% CI)	16 (9 to 25)	8 (6 to 11)	7 (4 to 12)	8 (4 to 15)	9 (5 to 15)
Unadjusted HR (95% CI)	Referent	0.52 (0.30 to 0.89)	0.46 (0.24 to 0.90)	0.50 (0.23 to 1.07)	0.59 (0.31 to 1.14)
Age and sex adjusted HR (95% CI)	Referent	0.71 (0.41 to 1.23)	0.66 (0.33 to 1.29)	0.67 (0.31 to 1.44)	0.79 (0.41 to 1.52)
PD-adjusted HR (95% CI)	Referent	0.51 (0.24 to 1.06)	0.45 (0.19 to 1.05)	0.47 (0.19 to 1.20)	0.57 (0.26 to 1.27)
Gastro-oesophageal cancer					
Number	12	20	8	5	7
Incidence rate per 10 000 patient-years (95% CI)	10 (5 to 18)	4 (2 to 6)	4 (2 to 7)	4 (1 to 9)	4 (2 to 8)
Unadjusted HR (95% CI)	Referent	0.35 (0.17 to 0.73)	NR	NR	NR
Age and sex adjusted HR (95% CI)	Referent	0.51 (0.24 to 1.05)	NR	NR	NR
PD-adjusted HR (95% CI)	Referent	0.59 (0.23 to 1.52)	NR	NR	NR

NR, not reported (indicates fewer than 10 events in each cohort so comparative analyses were not performed).

ADA, adalimumab; ETA, etanercept; INF, infliximab; PD, propensity score stratified into deciles; sDMARD, synthetic disease modifying antirheumatic drug; TNFi, tumour necrosis factor inhibitors.

### Outcome following cancer diagnosis

**Figure 2:**
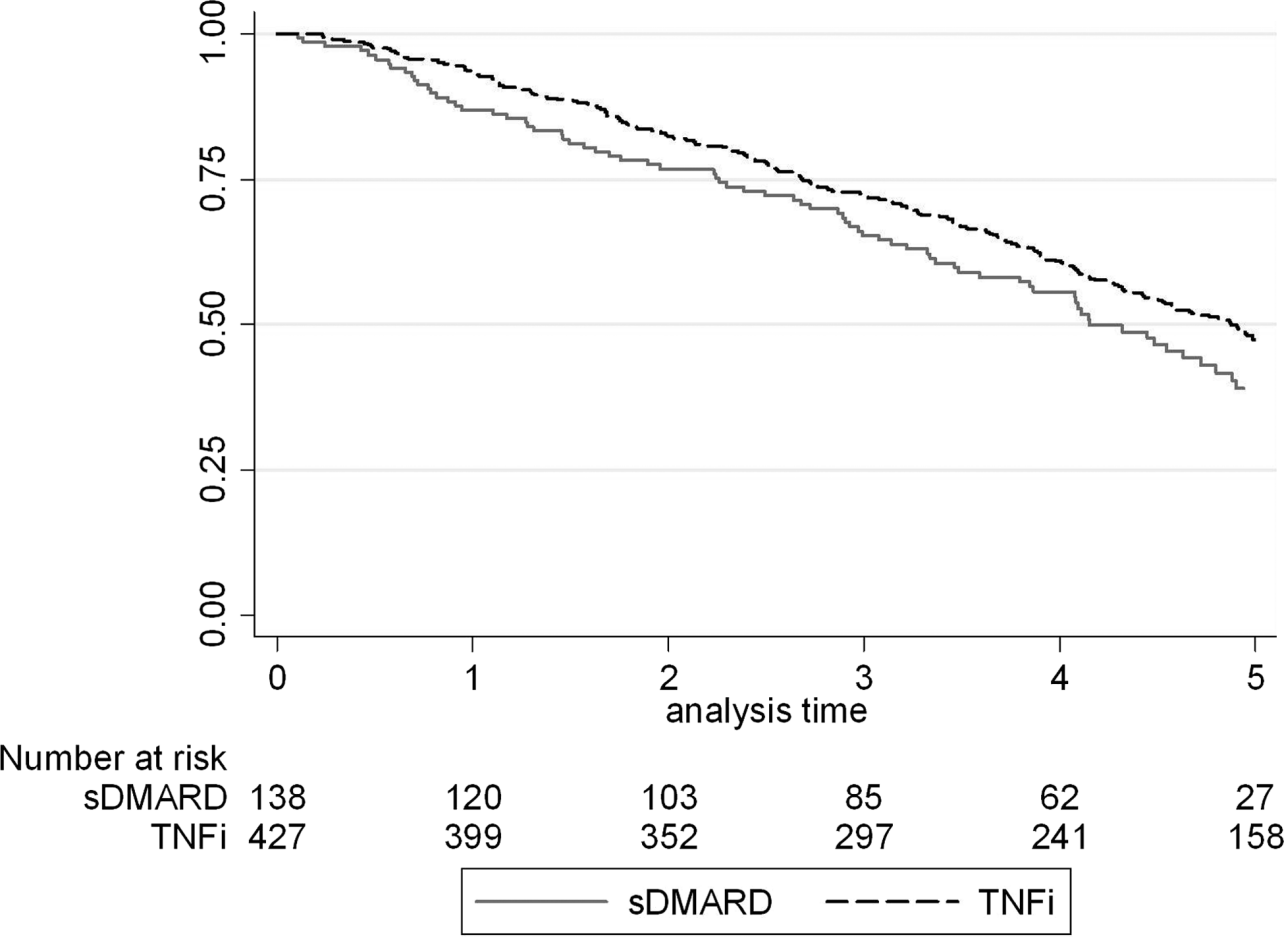
Kaplan-Meier survival curves for death following diagnosis with solid cancer in the BSRBR-RA. sDMARD, synthetic disease modifying antirheumatic drug; TNFi, tumour necrosis factor inhibitor.

Among the 563 patients with solid cancer, 309 patients died during subsequent follow-up; sDMARD 77 (57%); TNFi 232 (54%). Mortality was similar between the two cohorts and approximately linear (figure 2). For patients who died, the median survival time from date of cancer diagnosis was 118 days (IQR 6–342). There was no difference in the age and sex-adjusted risk of death between the two cohorts (HR 0.90, 95% CI 0.70 to 1.17).

## Discussion

Our study found no statistically significant difference in the risk of solid cancers in patients treated with TNFi compared to those treated with sDMARDs only, after adjusting for confounders. This confirms the findings from other European biologics registers,^8^ ^9^ ^12^ and from observational studies in North America.^10^ ^11^ This study adds value to the existing knowledge base because it included longer exposure time and more cancers in the TNFi-treated cohort than the previous European studies,^8^ ^9^ ^12^ and more rigorous data collection methods than the American study.^11^ Data from the national cancer registries as well as from hospital records and patients were used to identify and verify cancers. The results were consistent following various adjustments and sensitivity analyses. There was also no evidence of change in risk of solid cancer with increasing exposure to TNFi. This is therefore the largest and most robust study to date of the relationship between TNFi exposure and the risk of solid cancer in patients with RA.

The size of the BSRBR-RA dataset means that this analysis had sufficient power to investigate the RR of cancer for individual TNFi compared to sDMARD. The power to detect a 50% increased risk compared to sDMARD was 99% for ETA, 96% for INF and 98% for ADA. No difference in risk was found, and the 95% CI for each drug did not include a relative increased risk of more than 20%. Although the Swedish biologics register has reported no increased risk of cancer for individual TNFi, their analysis was not sufficiently powered to rule out clinically important differences: for example, the RR for ADA versus sDMARD was 1.32 (95% CI 0.87 to 1.98).^8^

The most frequently reported cancer sites were lung, breast, colorectal, female reproductive and gastro-oesophageal. There was no difference in the risk of either lung or gastro-oesophageal cancer between the cohorts. The site-specific results align well with some,^9^ ^18^ but not all^11^ ^12^ existing evidence. Of note was a non-significant reduction in the observed risk of breast and colorectal cancer, also observed in the German and Swedish biologic registers.^9^ ^18^ A recent publication from the BSRBR-RA reported that the rates of both breast and colorectal cancers in the sDMARD cohort were the same as in the general population (standardised incidence ratios 1.07 (95% CI 0.72 to 1.52) and 0.96 (95% CI 0.56 to 1.54) respectively),^19^ in contrast to other biologic-naïve cohorts in which reduced risks have been reported.^18^ ^20–22^ The signals for a reduced RR of breast cancer could reflect either unmeasured differences in subjects selected for TNFi or sDMARDs, or a true protective effect of the drug. TNF, within the microenvironment of breast cancer, has been shown to be associated with increased tumour invasiveness and poor prognosis,^23^ and so it is plausible that blocking the effects of TNF may slow or prevent the progression of breast cancer. However, TNF may inhibit breast cancer cell adhesion and proliferation.^24^ Etanercept has been trialled in the treatment of advanced metastatic breast cancer,^25^ although no objective disease responses were seen.

One reason for observing a reduced risk of breast cancer and colorectal cancer following TNFi might be screening prior to starting therapy, thus excluding cancers that would otherwise have been diagnosed during follow-up. However it is not routine practice to recommend such screening and this seems an unlikely explanation. Non-steroidal anti-inflammatory drugs (NSAIDs) have also been linked to reductions in breast^26^ ^27^ and colon cancer.^28^ However, data concerning ongoing NSAID use in this cohort were not captured and thus could not be accounted for in our analyses. In contrast to this study, a Danish study reported an increased risk of colon cancer in patients treated with TNFi versus unexposed patients (HR 3.52, 95% CI 1.11 to 11.15).^12^ They hypothesised that this may be due to the TNFi cohort being more sedentary.

No statistically significant difference in overall survival following cancer diagnosis was observed between the cohorts. A study from the Swedish biologics register has addressed this question previously.^29^ They found no difference in the risk of dying between 302 RA patients who developed cancer while being treated with TNFi and 586 biologic-naïve RA matched controls who developed cancer (HR for TNFi 1.1, 95% CI 0.8 to 1.6). Although the BSRBR-RA did not collect information on tumour stage at the time of diagnosis, the Swedish study found tumour stage at presentation to be largely similar between the groups, although the proportion of late presentations was higher in the control cohort (29% vs 20% stage IV), suggesting there may be a degree of surveillance bias in TNFi patients.

An ‘ever-exposed’ to TNFi model was selected as the primary exposure definition for TNFi because it was hypothesised that any effect of TNFi on cancer risk would be long-lasting and may operate in the latent period of a cancer. This analysis model means that patients in the TNFi cohort could have been exposed to other non-TNFi biologics prior to their incident cancer. However, alternative drug exposure models did not alter the findings.

Further complexities in the possible relationship between TNFi therapy and cancer risk exist that could not be fully accounted for in this analysis. It is possible that the overall finding of no difference in the relative incidence of solid cancer between the two cohorts was the result of risks acting in opposite directions for different cancer sites at different stages in the latent phase. Analysis by site, however, showed all four of the most common malignancies to have lower rates in the TNFi cohort compared to the sDMARD cohort.

The strengths of the study include the large size of the BSRBR-RA and detailed prospective collection of data relating to both drug exposure and outcome. Furthermore, patients registering with the sDMARD cohort were required to have active RA and be treated with sDMARDs, making them as similar as possible to the TNFi cohort. The broad inclusion criteria of the register mean that the results are more generalisable than those for RCTs. Linkage with the national cancer agencies minimised potential for bias in reporting between cohorts.

The weaknesses of the study are those of any observational study. The study findings reflect the way in which British rheumatologists selected patients for treatment with TNFi. At the time of the study, UK national guidelines listed cancer within the previous 10 years as a contraindication to TNFi.^14^ We also excluded patients with a prior cancer, meaning that these results cannot be extrapolated to patients with a previous cancer, who have been reported on previously.^30^ The proportion of missing baseline data was low. To minimise bias introduced by missing baseline data, multiple imputation was used. Response rates to follow-up questionnaires were excellent; less than 1% of patients in each cohort had no returned consultant follow-up. Unmeasured confounding (eg, differences in alcohol consumption) and channelling bias remain possible since subjects were not randomised to receive TNFi. Patients considered to be at high risk for developing cancer may have been preferentially recruited to the sDMARD cohort.

## Conclusions

In this registry, there was no difference in the overall risk of solid cancer in patients with RA treated with TNFi, or for any of the individual TNF inhibitors, compared to sDMARDs in the first 5 years of treatment. There was no evidence of change in risk of solid cancer with increasing exposure to TNFi. There was no difference in mortality following cancer after treatment with TNFi.

## Supplementary Material

Web supplement
