# Supplementary material for: Risk of solid cancer in patients exposed to anti-tumour necrosis factor therapy: results from the British Society for Rheumatology Biologics Register for Rheumatoid Arthritis
Source: Ann Rheum Dis. 2014 Mar 31;74(6):1087–93. doi: 10.1136/annrheumdis-2013-204851 (PMC4431340; doi:10.1136/annrheumdis-2013-204851)
Supplement: Web supplement [file annrheumdis-2013-204851-s1.pdf]

ONLINE-ONLY SUPPLEMENTARY MATERIAL

Statistical methodology supplementary information

Multiple imputations were performed in Stata version 12.1 using the ICE command. Missing data were present in the following variables: smoking, ethnicity, RA disease duration, baseline HAQ, and baseline DAS28 score (Table S1). The imputation model was constructed separately for the sDMARD and TNFi cohorts. Age, sex, disease duration, baseline HAQ, baseline DAS28 score and separate components (28 swollen joint count, 28 tender joint count, patient global visual analogue score and ESR), baseline steroid exposure, baseline non-steroidal anti-inflammatory exposure, prior sDMARD use, smoking status, entry year, co-morbidity, prior cancer, ethnicity, height and weight were all included as predictors within the imputation model. The outcome (solid cancer) was also included in the model. Twenty imputation cycles were performed and the resulting data were analysed using Rubin's rules with the MIM command.

Table S1. Missing baseline data

**Variable; N missing (%)**	**sDMARD**<br><br>**N=3249**	**TNFi**<br><br>**N=11767**
Disease duration	22 (1)	86 (1)
HAQ score	680 (21)	581 (5)
DAS 28 score	52 (2)	109 (1)
Smoking	15 (0)	76 (1)
Ethnicity	728 (22)	1635 (14)

Table S2. Unvariate analysis of individual covariates on risk of cancer and on risk of TNF in association with cancer

	**Hazard ratio (95% CI) for covariate**	**Hazard ratio (95% CI) for TNFi**
Unadjusted		0.70 (0.58, 0.85)
Age (per year)	1.06 (1.05, 1.07)	0.90 (0.74, 1.09)
Sex (Male referent)	0.71 (0.59, 0.85)	0.71 (0.58, 0.86)
Ethnicity (Non-white referent)	2.56 (1.21, 5.41)	0.70 (0.58, 0.85)
Smoking (Current smoker referent)		
Ex-smoker	0.96 (0.79, 1.17)	
Never smoked	0.47 (0.38, 0.59)	0.71 (0.58, 0.86)
Comorbidity: (Nil referent)		
1 comorbidity	1.24 (1.02, 1.50)	
2 comorbidities	1.35 (1.06, 1.72)	0.72 (0.59, 0.87)
≥3 comorbidities	1.91 (1.39, 2.61)	
Entered study before June 2004	0.92 (0.77, 1.09)	0.72 (0.59, 0.88)
Disease duration (per year)	1.01 (1.00, 1.02)	0.67 (0.55, 0.82)
Disease activity (per unit DAS28)	1.11 (1.02, 1.21)	0.61 0.49, 0.76)
Disability (per unit HAQ)	1.19 (1.03, 1.38)	0.64 (0.52, 0.79)
Baseline corticosteroids	1.13 (0.95, 1.34)	0.68 (0.56, 0.83)
No. prior sDMARD: (≤3 referent) ≥4	1.20 (1.01, 1.43)	0.66 (0.54, 0.81)
Ever exposed to AZA	1.15 (0.93, 1.42)	0.68 (0.55, 0.83)
Ever exposed to CYC	2.04 (1.30, 3.19)	0.68 (0.56, 0.84)

Table S2 shows first the associations between candidate confounders and the outcome (first solid cancer), irrespective of treatment group. This demonstrated an association between increasing age and solid cancer and male sex and solid cancer, as expected. Further associations were observed with smoking, comorbidity and markers of RA severity. The final column reports the effect of each baseline covariate on the estimated treatment effect. Age and DAS28 score had the greatest effect on the estimated effect of anti-TNF on risk of solid cancer, indicated by the change in HR for anti-TNF from 0.70 to 0.90 and 0.61 respectively (Table S2). None of the other confounders altered the HR by more than 10% in univariate adjustment. None the less, all the *a priori* selected confounders were included in the analysis since interactions and non-linear associations may have existed.

Components of the PS model were: age, sex, ethnicity, smoking status, co-morbidity, RA duration, DAS28, HAQ, steroid exposure, number of prior sDMARD (≤3 versus 4 or more), prior exposure to AZA and prior exposure to CYC at baseline. In addition, during construction of the PS model, interaction terms between HAQ and CYC; age and DAS28; age and disease duration; prior sDMARD exposure and smoking; sDMARD exposure, smoking and disease duration; and sDMARD exposure, smoking and DAS28 were added. The expected bias was <2% for each variable. The overall expected bias was -0.7%.

Table S3. Subtypes of verified solid cancers in the BSRBR-RA

**Cancer site: N(%)**	**sDMARD N=136**	**TNFi N=427**
Lung	40 (29)	103 (24)
Female breast	22 (16)	73 (17)
Colorectal	19 (14)	43 (10)
Female reproductive cancers	4 (3)	42 (10)
Gastro-oesophageal	12 (9)	20 (5)
Urinary/renal tract	7 (5)	29 (7)
Male reproductive cancers	4 (3)	23 (5)
Lip to pharynx	3 (2)	16 (4)
Melanoma	7 (5)	7 (2)
Pancreas	2 (1)	12 (3)
Nervous system	3 (2)	11 (3)
Hepatobiliary	3 (2)	5 (1)
Endocrine	0 (0)	5 (1)
Mesothelioma	1 (1)	2 (0)
Small bowel	0 (0)	4 (1)
Peritoneal	1 (1)	2 (0)
Sinus	0 (0)	3 (1)
Anal	0 (0)	2 (0)
Metastasis, no primary site	2 (1)	6 (1)
No site	6 (4)	19 (4)
